# Primary Cilium Is Involved in Stem Cell Differentiation and Renewal through the Regulation of Multiple Signaling Pathways

**DOI:** 10.3390/cells10061428

**Published:** 2021-06-08

**Authors:** Sila Yanardag, Elena N. Pugacheva

**Affiliations:** 1 Department of Biochemistry, School of Medicine, West Virginia University, Morgantown, WV 26506, USA; sy0016@mix.wvu.edu; 2 West Virginia University Cancer Institute, School of Medicine, West Virginia University, Morgantown, WV 26506, USA

**Keywords:** primary cilia, stem cells, cancer stem cells, signaling, differentiation, Notch, Wnt, TGF, mTOR

## Abstract

Signaling networks guide stem cells during their lineage specification and terminal differentiation. Primary cilium, an antenna-like protrusion, directly or indirectly plays a significant role in this guidance. All stem cells characterized so far have primary cilia. They serve as entry- or check-points for various signaling events by controlling the signal transduction and stability. Thus, defects in the primary cilia formation or dynamics cause developmental and health problems, including but not limited to obesity, cardiovascular and renal anomalies, hearing and vision loss, and even cancers. In this review, we focus on the recent findings of how primary cilium controls various signaling pathways during stem cell differentiation and identify potential gaps in the field for future research.

## 1. Structure and Function of Primary Cilia

Primary cilia are protrusions built on top of the centriole (basal body) and are present in most stem cells [1,2]. The core of the primary cilia (axoneme) is composed of microtubules connected to the basal body, which is anchored to the cell membrane via the distal appendages. The axoneme is arranged in a 9 + 0 fashion, i.e., nine outer microtubule doublets and no central singlets [2,3]. Each doublet is composed of a complete A-tubule and a partial B-tubule. Retrograde transport, where the cargo is transported from the tip to the base of the cilia, takes place on the A-tubule and is carried out by dynein proteins [4,5].

**Figure 1:**
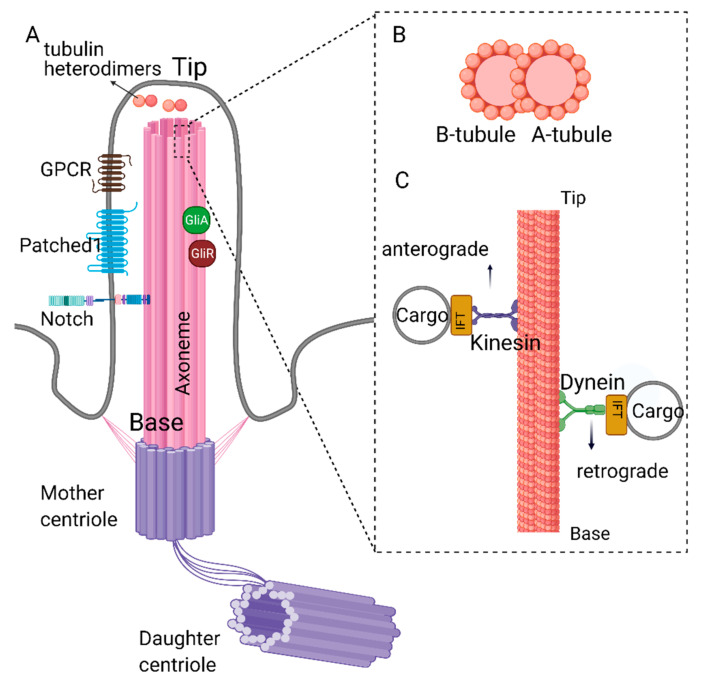
Structure of primary cilia. (**A**) Primary cilium is nucleated from the mother centriole of the basal body. Axoneme consists of 9 microtubule doublets formed by α and β tubulin heterodimers. (**B**) Cross-section of complete A- and partial B-tubules. (**C**) Dynein and kinesin proteins carrying cargo on the axoneme. Retrograde movement (from tip to the base) is carried out by dynein proteins, whereas kinesin proteins carry out anterograde movement (from base to the tip). Kinesin and dynein proteins are essential for the assembly and disassembly of the primary cilium as they carry building blocks or the depolymerizing agents along the axoneme. Created with BioRender.com.

Similarly, anterograde trafficking happens on the B-tubule, and the proteins that aid this movement are called kinesins. The building blocks of the A and B tubules (αβ tubulin heterodimers) are transported to the tip of the cilia by kinesins (KIF3A/B) and intraflagellar trafficking particles (IFTs74, 81) [6,7]. The αβ tubulin heterodimers move along the axoneme of *Chlamydomonas* *reinhardtii* primarily through passive diffusion, rather than the active transport by the IFT particles [8]. Additionally, IFT particles involved in cargo transport include but are not limited to IFT20, IFT74, and IFT88 [6,7,9,10,11,12] (Figure 1). For a detailed review of IFT particles and ciliary transport, see reference [13]. The deletion/depletion of IFT88/Polaris or KIF3 kinesins is often used to disable cilia construction, rendering cells non-ciliated. The axoneme is surrounded by the ciliary membrane and forms the ciliary pockets connected to the cell membrane (Figure 1). Although the ciliary membrane is an extension of the cell membrane, its composition is different to support complex signal transduction. The ciliary membrane contains phosphatidylinositol 4-phosphate (PI(4)P) instead of phosphatidylinositol 4,5-bisphosphate (PI(4,5)P2), which is present in the cellular membrane [14,15]. The ciliary membrane harbors higher concentrations of sphingolipids and ganglioside enriched lipid rafts [16,17].

Primary cilium biogenesis is a cell cycle-dependent event as the centrosomes are the major players in both primary cilium biogenesis and mitosis. During the G1/G0 phase, primary cilium is nucleated from the plus end of the mother centriole. Upon transitioning to the S/G2 phase, cilia disassembly and resorption are initiated [18]. This process is tightly controlled by the activity of various proteins such as Aurora A kinase, HDAC6, Nedd9, etc. [18,19,20,21,22]. Based on its position and structure, the primary cilium is a major signaling hub residing at the crossroads of multiple processes.

## 2. Primary Cilia and Signal Transduction

Primary cilia in stem cells function as a signaling hub for transducing signals of many pathways. These pathways include receptor tyrosine kinase (RTK), transforming growth factor-β (TGF-β), G-protein coupled receptor (GPCRs), Hedgehog (Hh) Wingless/int (Wnt), Notch, and mechanistic target of rapamycin (mTOR) [23,24,25]. Moreover, due to its physical build-up on top of the centriole, the cilia function as negative regulators of cell proliferation and are often found in quiescent cells, including stem cells. The disassembly of the cilium in response to growth factors or stem cell niche cues leads to the release of the mother centriole, a vital step for forming mitotic spindles and initiation of mitosis [19,21,26]. Defects in primary cilia dynamics (assembly/disassembly) or complete loss of cilia in stem, progenitors, or terminally differentiated cells have been associated with many diseases collectively known as ciliopathies [27,28,29]. There are at least 35 identified ciliopathies, including polycystic kidney disease, cone-rod dystrophy, hydrocephaly, and medulloblastoma, to name a few. Additionally, there are over 150 established and over 200 potential ciliopathy-related genes. Details of the ciliopathy diseases, genes and treatment options are reviewed by Reiter et al. [27], Mitchison et al. [30], McConnachie et al. [31], and Duong Phu et al. [32].

## 3. Stem Cell Biology and Classification

Stem cells are the focus of many scientific explorations ranging from neurodegenerative diseases to cancer, due to their ability of self-renewal and capacity to differentiate into many types of cells. Stem cells have primary cilium, but the role of cilium in their biology is not fully defined, especially in adult stem cells. The classification of stem cells depends on their origin during development. Totipotent cells are formed by the first several divisions of the fertilized egg. They have the highest differentiation potential. At 3–5 days post fertilization, pluripotent embryonic stem cells (ESCs) are formed. They make up the embryo’s inner cell mass (ICM) and differentiate into endoderm, mesoderm, and ectoderm [33]. Primary cilia are a general feature of ESCs and essential signaling hubs for differentiation and self-renewal pathways [34]. The role of primary cilia in embryonic development has been extensively studied using several model organisms, and the most significant outcomes are reviewed here.

## 4. Primary Cilia in Embryonic Development: ECSs

The primary cilium is essential for healthy and complete embryonic development. Primary cilia first appear on the epiblast cells at E6 and are present on all derivatives of the epiblast but absent on trophoblasts and visceral endoderm ESCs [35]. Mouse embryos with the homozygous knockout of KIF3A, KIF3B, or IFT88 lack primary cilia in the primitive node while their wild-type counterparts are ciliated. These embryos show significant developmental defects by the time they reach E8-E9. These defects include incorrect left–right asymmetry (situs-inversus), cardiac loop inversion and pericardiac sac ballooning, edema around the heart, and neural tube closure defects [36,37,38,39]. These developmental defects and lethality are attributed to the lack of primary cilia in the nodal cells or the cells that arise from them, including neuroectoderm cells, giving rise to neural tube and neural crest [36,37,38,39,40]. Ciliary depletion via conditional KIF3A knockout in neural crest cells causes defects in hindbrain development but does not cause lethality [40]. These results indicate that the defects induced by homozygous deletion of KIF3A, KIF3B, or IFT88 in mouse embryos occur before forming neural crest cells. However, the differentiation capabilities of cilia-depleted nodal cells into neurectoderm and other germ layers remain to be investigated. Without primary cilium, cellular signaling pathways crucial for development are interrupted or dysfunctional. This idea is further supported in zebrafish embryos during development. In zebrafish, whole-body knockout of IFT88, KIF3A, FSD1, or PKD2 causes developmental defects such as body curvature and abnormal spawn extension but does not cause lethality [41,42]. Unlike mice, zebrafish embryos can survive past gastrula (5 h post-fertilization) [41,43] and develop to term. Similar to reduced Hh signaling in mouse embryos [38], mutant zebrafish embryos also exhibit lower levels of Hh signaling [41], which appears to be critical at this stage of development.

## 5. Primary Cilia in Organ Development/Maintenance: Adult Stem Cells and iPSCs

Neural Progenitors (NPs) and Neural Stem cells (NSCs) which are differentiated from ESCs, also possess primary cilia and require it for the differentiation of multiple types of brain cells in adult organisms [44,45]. The ESCs features are controlled by distinct transcription factors [46,47]. Oct4 is one of the major transcription factors stably and uniformly expressed throughout the maturation of stem cells [48]. Another critical transcription factor for the cell-fate specification is Nanog, which regulates ESC pluripotency through multiplex interactions with other transcription factors such as Oct4, Sox2, and Klf4 [49]. The Oct4, Sox2, Nanog, and Myc combination can reprogram terminally differentiated adult somatic cells into induced pluripotent stem cells (iPSC) [50]. The iPSCs have primary cilia [51], but the role of the primary cilium in the reprogramming of terminally differentiated cells is currently unknown. However, mechanical properties of cilia have been altered during reprogramming of fibroblasts to iPSCs [52]. Mesenchymal stem cells (MSCs) are multipotent stromal cells of mesodermal and neural crest origin. These cells are often used in tissue engineering. The MSCs derived from bone marrow, adipose tissue, muscle, or umbilical cord can differentiate into osteoblasts, chondrocytes, adipocytes, and myocytes [53,54]. Multiple studies have been conducted to examine the role of primary cilia in MSCs and their progenitors to produce differentiated cells. It was shown that primary cilia-dependent signaling is required for MSCs’ proliferation and pluripotency. The expression of mentioned above stem cell markers Oct4, Nanog, and Sox2, were downregulated after the deciliation [55].

Similarly, expressions of Oct4, Nanog, and Sox2 were increased upon inhibition of Aurora A kinase (AURKA) in adipose-derived MSCs isolated from obese mice while recovering the functional primary cilia [56,57]. These findings indicate the critical role of the primary cilium in regulating stemness in pathological and healthy conditions. This review focuses on the analysis of the signaling pathways involved in the maintenance and differentiation of adult and embryonic stem cells impacted by primary cilia. We also identify potential gaps in the research for future directions.

## 6. Role of Primary Cilia in Wnt/β-Catenin Regulation and Stem Cell Biology

**Figure 2:**
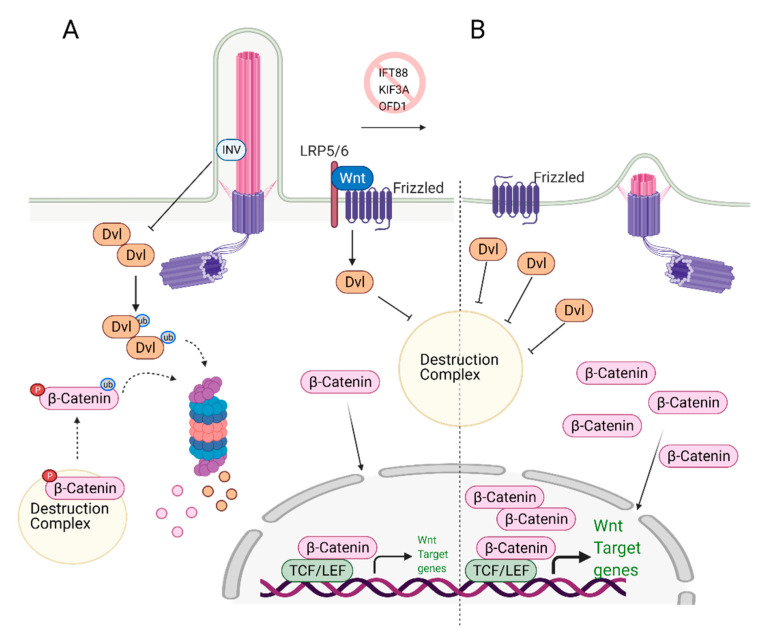
Role of primary cilia in Wnt/β-catenin regulation and stem cell biology. (**A**) Primary cilium keeps the expression of Wnt target genes in check. Upon binding of Wnt ligands to the receptors, Disheveled (Dvl) inactivates the destruction complex, causing release and translocation of β-catenin to the nucleus. In the nucleus, it serves as a transcription activator and initiates the expression of Wnt target genes. At the same time, excess Dvl is targeted to proteasomal degradation via ciliary-localized Inversin (INV), causing degradation of some of the β-catenin. (**B**) In the absence of primary cilium, increased cytoplasmic localization of Dvl is observed. This causes increased levels of cytoplasmic and nuclear b-catenin, leading to overexpression of Wnt/β-catenin target genes. Created with BioRender.com.

Wnt signaling plays a critical role in MSCs and ESCs maintenance and differentiation [58,59]. Signaling pathways controlled by Wnt can be categorized as canonical (β-catenin pathway) and non-canonical (planar cell polarity and Wnt/Ca2+ pathways) and were previously reviewed in detail by Komiya et al. [60] and Clevers and Nusse [61,62]. In the canonical Wnt signaling pathway, Wnt ligands (Wnt1, Wnt2 Wnt2b, Wnt3, etc.) [63] bind to the G-protein coupled receptor Frizzled (Fzd) and Low-density lipoprotein receptor-related protein (LRP) 5/6, initiating a cascade of events that results in the stabilization and nuclear translocation of transcription factor β-catenin and transcription of Wnt-responsive genes (Figure 2A) [64,65,66,67]. The non-canonical Wnt signaling pathway is β-catenin independent. In the planar cell polarity (PCP) pathway, the binding of Wnt ligands to their receptors activates the downstream effector c-Jun N terminal kinase (JNK), which controls the cytoskeleton. In the Wnt/Ca^2+^ pathway, ligand-bound Fzd receptors and co-receptor Receptor Tyrosine Kinase-like Orphan Receptor-1/2 (ROR1/2) trigger the activation of phospholipase C (PLC), which in turn signals for Ca^2+^ release from intracellular calcium stores. Calcium release causes transcriptional activation of genes controlling cell fate and migration while inhibiting β-catenin/TCF/LEF mediated transcription.

Wnt Signaling and Primary Cilia intersection: Primary cilia play an essential role as regulators of Wnt signaling in various stem cells. Knockdown of the genes that cause cilia loss, such as IFT88 or KIF3A, leads to defects in the differentiation potential of the stem cells. Specifically, loss of cilia via depletion of IFT88, leads to an increase in Wnt5a/β-catenin levels and defects in adipogenic differentiation, as determined by the reduced expression of the adipogenic markers PPARγ and CEPBα [68]. These results imply that primary cilia suppress Wnt/b-catenin expression, which is needed to induce adipogenic differentiation. Thus, both canonical Wnt and non-canonical Wnt/Ca^2+^ pathways are activated when cilia are lost [68]. The deciliation caused by KIF3A knockout in mouse embryonic stem cells (ESC) and mouse embryonic fibroblasts (MEFs) resulted in increased Wnt/β-catenin activity compared to the wild-type, ciliated controls [69]. The authors further show that similar results were obtained when deciliation was achieved via IFT88 or OFD1 knockdown. The non-ciliated ESCs and MEFs cells have more stabilized and elevated levels of nuclear β-catenin regardless of Wnt3a/5a stimulation (Figure 2B). Interestingly, the amount of phosphorylated disheveled (Dvl), a key regulator of Wnt signaling pathways, was found to be higher in these cells too. These results suggest that primary cilia mediate Wnt/β-catenin signaling in the regulation of embryonic stem cells [69]. In this context, cilia serve as a negative regulator of Wnt activation. It should be noted that the reporter assay results of a similar study conducted by another group did not lead to the same conclusion [70]; however, nuclear vs. cytoplasmic levels of β-catenin or Dvl as well as the depletion efficiency of IFT88 and IFT172 were not reported in this study.

On the other hand, KIF3A knockdown in adult dental follicle stem cells (DFC) and dental pulp stem cells (DPC) results in inhibited differentiation of these cells into osteoblasts. However, when the cells are supplemented with recombinant Wnt3a, expression of the differentiation markers were recovered, indicating that halted osteogenic differentiation in KIF3A knockdown cells is due to primary cilium mediated impairment in Wnt/β-catenin signaling [71]. Similarly, conditional KIF3A depletion in osteoblasts of newborn mice resulted in reduced bone formation and caused osteopenia. These mice also exhibited decreased Ca^2+^ deposition in the extracellular matrix, reduced Wnt/β-catenin signaling, and reduced Hh signaling. As a result of impaired signaling activities, a shift from osteogenesis to adipogenesis was observed when KIF3A is conditionally depleted in osteoblasts postnatally [72]. The change from one signaling axis to another is often associated with deciliation, as demonstrated in IFT88-deficient midbrain dopaminergic neuron progenitors [73]. In this case, a deficit of Shh signaling caused by lack of cilia leads to activation of Wnt, which is used later on for neuron development. The convergence of Shh and Phosphoinositide (PI3K/Akt/mTORC1) signaling at cilia transition zone function was also noted in INPP5E embryos [74]. These results show that the primary cilium-mediated signaling cascade is not only needed for correct stem cell differentiation but is also crucial to maintain proper cellular fate. Furthermore, these findings highlight the context-dependency of ciliary involvement in the regulation of signaling pathways and.

## 7. Role of Primary Cilia in TGF-β Regulation and Stem Cell Biology

**Figure 3:**
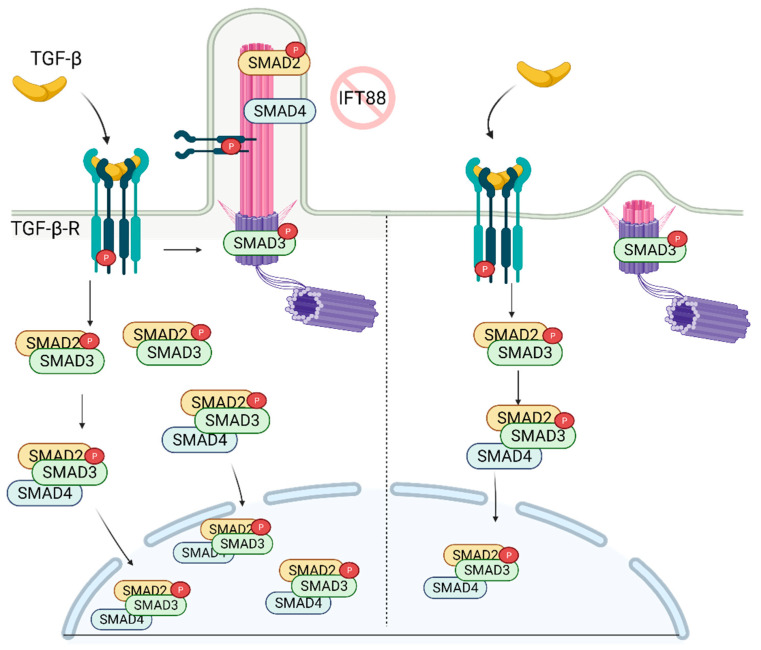
TGF-β signaling is regulated by primary cilia. In the presence of primary cilia, p-TGF-β-RI, p-SMAD2, and SMAD4 are localized on the cilia, while p-SMAD3 is localized on the ciliary base. Stimulation of the receptor with TGF-β ligand results in increased nuclear localization of active SMAD2/3/4 complex. In the absence of cilia, while p-SMAD3 is still localized on the ciliary base, its nuclear localization is diminished as well as the abundance of SMAD2/3 complex in the cell. Created with BioRender.com.

The recruitment of mesenchymal stem cells (MSCs) is a crucial process in developing, maintaining, and repairing tissues. TGF-β is a potent chemokine essential for the recruitment of MSCs and plays a critical role in stem cell proliferation and differentiation [75]. The binding of TGF-β to the receptor (TGF-β-R) on the cellular membrane initiates the cascade of signaling events in the cytoplasm resulting in the activation/translocation of SMADs (SMAD2, SMAD3, and SMAD4) to the nucleus (Figure 3). The SMADs then aid in the transcription of a wide range of TGF-β dependent genes involved in the regulation of stemness [76].

Dysregulation of TGF-β signaling or cilia has been linked to several skeletal pathologies. In the recent report, it was shown that recruitment of MSCs involved in bone formation relies on the proper construction of the primary cilium [77], which is needed for the activation of TGF-β. Active SMAD2 (p-SMAD2), p-SMAD3, and SMAD4, along with p-TGF-β-R, localize on the ciliary axoneme and base (Figure 3). Importantly, knockdown of IFT88 (deciliation) reduced levels of p-SMAD2/3 following TGF-β stimulation. This correlates with the diminished levels of nuclear p-SMAD2/3 [77] (Figure 3). Moreover, depletion of IFT88 in MSCs rendered cells less chemotactic, highlighting the key role of the primary cilium in the regulation of skeletal pathogenesis. These findings suggest that primary cilia are required for optimal signal transduction/activation of bone marrow MSCs following stimulation with TGF-β.

The lack of primary cilium during embryogenesis results in the defective vasculature and heart formation [78]. TGF-β plays an essential role in heart development, with primary cilia present in the stem and differentiated progenitors. During cardiomyogenesis, TGF-β-RI and II, SMAD2/3/4 upregulation, and SMAD7 (inhibitor of SMAD2/3) downregulation were observed. The TGF-β-RI/II and SMAD2/3/4 were localized at the ciliary base before they translocate to the nucleus, suggesting the regulation and control of TGF-β signaling through the primary cilia [23]. However, this observation is only correlative. Additional experiments, e.g., depletion of IFT88 or KIF3A during or before cardiomyocyte differentiation, are needed to demonstrate the active role of primary cilia in differentiation.

TGF-β stimulation causes loss or shortening of primary cilia in osteoblasts. The TGF-β inhibits the maturation and differentiation of osteoblasts through the inactivation of bone morphogenic protein (BMP) 2 and BMP7, major proteins that induce osteoblast differentiation. Interestingly, the HDAC inhibitor can rescue cells from TGF-β-mediated inhibition of BMP2 and BMP7 signaling. It was previously shown that HDAC6 inhibitors inhibit primary cilium disassembly [18]. When the osteoblasts are co-treated with TGF-β and the HDAC6 inhibitor, tubacin, differentiation is restored as documented by the increased alkaline phosphatase activity of the osteoblasts-positive regulation of matrix mineralization. Collectively, these results indicate that the primary cilium is a key regulator of the TGF-β and BMP signaling during osteoblast maturation. Restoration of structure and function of the primary cilia might be a viable approach to use in the clinic as a therapeutic intervention to treat patients suffering from diabetes [56,57] or osteoarthritis as these chronic diseases exhibit elevated levels of TGF-β and bone resorption [79].

## 8. Role of Primary Cilia in mTOR Regulation and Stem Cell Biology

**Figure 4:**
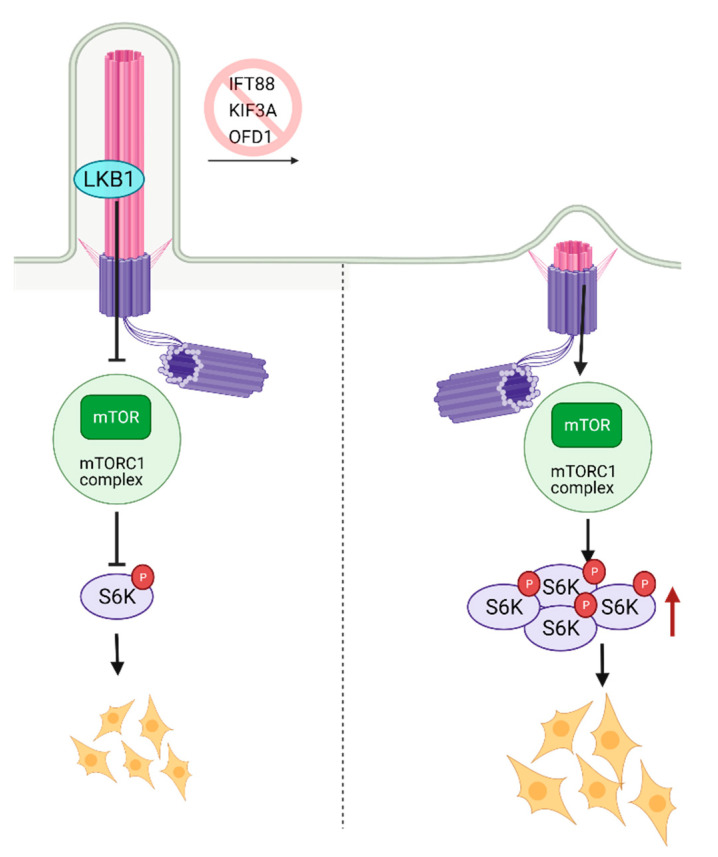
Regulation of mTOR signaling through primary cilia. Ciliary localized LKB1 inhibits mTOR, and keeps mTORC1 activity at a lower level. Inhibition of primary cilia formation results in increased mTORC1 activity, increased levels of active S6K, and larger cell size. Created with BioRender.com.

The mammalian target of Rapamycin (mTOR) is a serine-threonine kinase that is ubiquitously expressed in mammalian tissue [80,81,82]. mTOR acts through mTORC1 and mTORC2 complexes to assess growth factors and nutrient availability and is a fundamental regulator of metabolism and survival [81,82]. The metabolic balance between glycolysis and mitochondrial oxidative phosphorylation is essential for stem cell’s self-renewal and differentiation capacity [83]. High glycolytic metabolism is needed to maintain pluripotency but, as stem cells mature and differentiate, they switch towards oxidative phosphorylation [84] in an mTOR-dependent manner. Inhibition of mTOR with rapamycin has been shown to promote somatic cell reprogramming to iPSCs [85]. The activity of mTOR was shown to be cilia-dependent. One of the first studies that documented the role of primary cilia in mTOR signaling reported activation of this pathway due to a deficiency in expression of the OFD1 gene (oral-facial digital syndrome-1) in mouse kidney tissue [86]. OFD1 is a centrosome-associated protein involved in IFT88 recruitment to the primary cilia and the primary cilia biogenesis. Since the homozygous deletion of OFD1 in the mouse embryo is lethal [86], kidney-specific conditional OFD1 depletion was analyzed. The authors had found that OFD1 depletion leads to the lack of ciliation and increased mTOR activity [86], indicating that cilia are negative regulators of mTOR activity (Figure 4). The hyperactivation of mTOR reduces the self-renewal of stem cells; thus, balanced mTOR activity is pivotal for development. The homozygous mTOR depleted mouse embryos fail to grow past embryonic day 6 (E6). Since complete depletion of mTOR was embryonically lethal, functionally impaired mTOR (kinase-dead) was introduced to the blastocytes resulting in smaller cell size and slower proliferation. Complementary to these findings, it was shown that depletion of primary cilia in mouse kidney cells causes an increase in cell size [87]. The mTORC1 pathway is negatively regulated by LKB1 kinase, which is localized in the primary cilia. Depletion of primary cilia resulted in activation of mTORC1 and its downstream target S6K [87] (Figure 4). LKB1 localization to the primary cilia is needed to inhibit CCL2 expression [88] and potentially exert its inhibitory function on mTORC1 and thus sustain a low level of mTORC1 activity needed for stem cell renewal and proliferation. Similarly, deciliation of the radial glial cells (RGCs), the progenitor cells which give rise to glia and neurons during embryonic development, via the deletion of KIF3A or IFT88, also resulted in activation of mTORC1 [89] (Figure 4). In addition to the increased mTORC1, RGC deciliation caused hydrocephaly, a common phenomenon with ciliopathy diseases.

Another aspect of mTOR signaling affecting stem cells is its role in autophagy, which is regulated by ciliogenesis [90]. It was shown that ciliation deficiency in mouse embryonic fibroblasts (MEFs) induced by knockdown of IFT20 caused downregulation of autophagy [90]. Similarly, inhibition of autophagy suppresses ciliogenesis [91]. It is tempting to speculate that deciliation-mediated increases in mTOR activity is the reason for downregulation in autophagy; however, mTOR inhibition in the deciliated cells did not return autophagy to the basal levels [90], suggesting that additional mTOR-independent regulation of autophagy by primary cilia exists.

## 9. The Role of Autophagy and Primary Cilia in Embryonic Stem Cells

The multiple proteins involved in the formation of autophagosomes (LC3, ATGL16, etc.) are located on the basal body, transition zone, or the primary cilium itself [90], indicating the potential involvement of primary cilium in autophagosome formation/activity.

**Figure 5:**
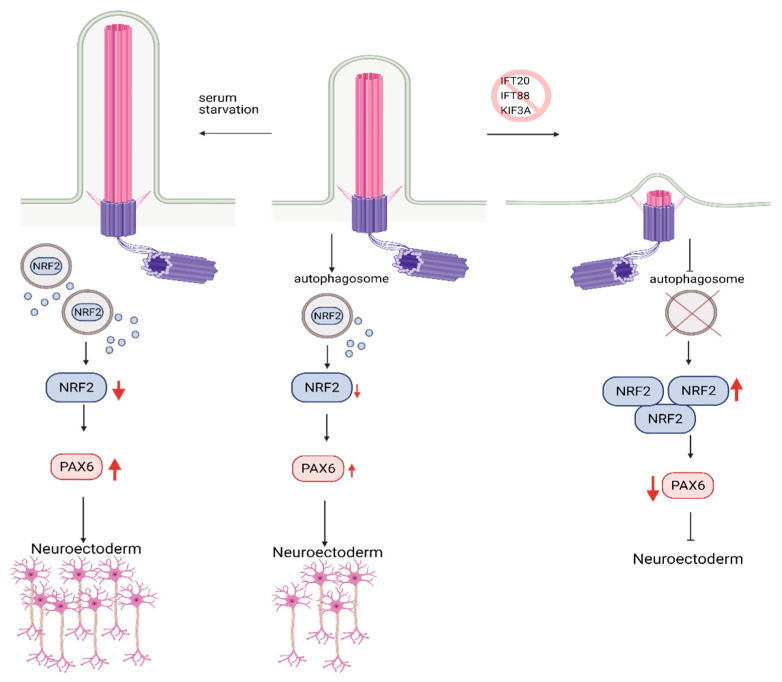
Role of Autophagy and primary cilia in embryonic stem cell lineage specification. Primary cilium promotes autophagosome formation in hESCs, keeping embryonic development in check via the controlled degradation of Nrf2. Inhibition of primary cilia formation results in increased levels of Nrf2 due to constrained autophagosome formation. Increased levels of Nrf2 reduce the expression of PAX6, neuroectoderm fate-determinant transcription factor, which in turn results in inhibition of hESC’s commitment to neuroectoderm lineage. Conversely, activation of primary cilia via serum starvation causes higher levels of PAX6 expression and promotes commitment to neuroectoderm lineage. Created with BioRender.com.

In agreement with this conclusion, the recent study in hESCs found that primary cilium is involved in fate/lineage determination during the early stages of embryonic development through the autophagosomal degradation of Nuclear factor erythroid 2-like (Nrf2). Nrf2 is a master transcription factor that plays a crucial role in mesendoderm (embryonic tissue layer that differentiates into mesoderm and endoderm) or neuroectoderm (embryonic tissue from which neuronal progenitors arise, which generates neurons and glial cells) lineage specification in hESCs [92]. Similar to the earlier studies, blockade of primary cilium formation via knockdown of IFT88, KIF3A, or IFT20 in hESCs halted autophagosome formation, inhibiting Nrf2 degradation. Nrf2 accumulation switched differentiation of hESCs from neuroectoderm towards mesendoderm lineage [92] (Figure 5). However, there was no differential expression of mTOR response genes in neuroectoderm and mesendoderm lineages. Thus, primary cilium-mediated lineage specification through autophagosome activation in an mTOR-independent. These findings highlight a novel role for primary cilium, bypassing mTOR in the regulation of autophagy. However, further studies are needed to delineate this process.

## 10. Role of Primary Cilia in Notch Regulation and Stem Cell Biology

Notch signaling is a conserved, intercellular communication pathway that regulates stem cell fate determination, differentiation, and self-renewal in adult tissues and embryonic development [93]. Interestingly, multiple Notch signaling components have been shown to localize to the primary cilium.

**Figure 6:**
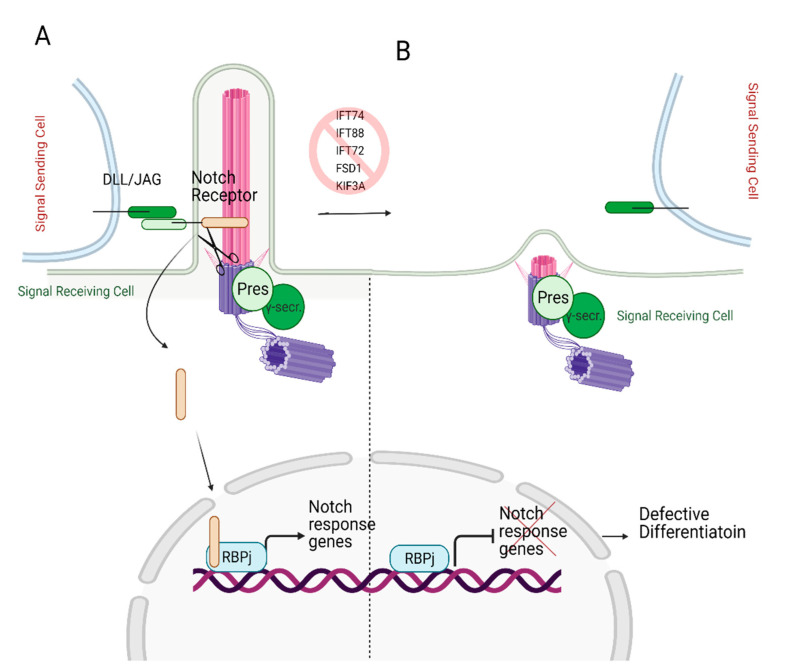
Role of primary cilia in Notch signaling. (**A**) Notch receptor localizes on the primary cilium. Following the binding of the Notch receptor to the ligands DLL/JAG, NICD is cleaved by presenilin (Pres), the catalytic subunit of γ-secretase, which localizes on the basal body. Cleaved NICD translocates to the nucleus. It serves as a transcriptional co-activator of the RBPj (Recombination signal Binding Protein for immunoglobulin kappa j region) to express Notch response genes. (**B**) In the absence of primary cilia, expression of Notch target genes is halted since Notch receptor cannot localize to the ciliary membrane and be cleaved by presenilin, resulting in defective differentiation. Created with BioRender.com.

A Notch signaling pathway requires two cells to participate: a signal sending cell and a receiving cell. Signal sending cells harbor the ligands for the notch signaling, transmembrane proteins called Delta-like (DLL) and Jagged (JAG). On the other hand, the receiving cells contain Notch receptors. Upon ligand binding, Notch receptor undergoes multiple cleavage cycles by γ-secretase, producing Notch IntraCellular Domain (NICD). The NICD then translocated to the nucleus [94,95,96], transactivating Notch target genes (Hes and Hey, myc, runx1, sox9) [42,97,98,99] (Figure 6A). The Notch-dependent transcription regulation is reviewed in depth in reference [100].

Ezratty and colleagues [101] first identified the involvement of primary cilium in the Notch signaling pathway in 2011 using keratinocytes and embryonic epidermis. Notch signaling is crucial for skin development as it triggers the differentiation of keratinocytes during early embryonic development [102]. The authors reported the co-localization of the Notch receptor on primary cilia and the presenilin, catalytic subunit of γ-secretase, on the basal body. The deciliation via knockdown of IFT88 or KIF3A in the mouse embryo skin resulted in reduced Notch receptor activity in differentiating embryonic epidermal cells, which lead to defective cell commitment (Figure 6B). However, defective cellular differentiation was partially restored by the expression of NICD. These findings suggest that Notch signaling requires intact and functional primary cilium to initiate the differentiation of the embryonic epidermis [101].

Similarly, stemness of fallopian tube adult stem cells is Notch/Wnt/cilia dependent [103]. The authors also noted that disruption of primary cilium via depletion or conditional knockout of IFTs, but not KIF3A, resulted in a hyper-proliferative phenotype. Since multiple signaling nodes often crosstalk, it is interesting to note that Notch signaling could activate Smo and Gli in a cilia-dependent manner in a Shh-independent fashion [104].

Similarly, Notch signaling is downregulated in cilia-impaired (knockout of cilia building genes FSD1, KIF3A, PKD2, and IFT88) zebrafish embryos compared to the wild type. The study by Liu et al. showed that primary cilium is needed for hematogenic endothelial cell (HE) differentiation through the regulation of Notch signaling. HE cells give rise to hematopoietic stem and progenitor cells (HSPC). Reduced expression of HSPC markers, RUNX1 and CYMB, was observed in cilia-impaired zebrafish embryos due to reduced protein levels of NICD [42]. These results suggest that Notch signaling acts downstream of the primary cilium to control the HE to HSPC differentiation in the zebrafish embryo. This could be through stabilizing the Notch receptor, as evident with the reduced levels of NICD protein in cilia-deficient cells. Another possible reason for reduced NCID levels could be deficient autophagosomal degradation of the protein in deciliated cells. The autophagosome inhibition in cilia-deficient cells may provide potential answers.

## 11. Primary Cilia in Cancer and Cancer Stem Cells

Interestingly, with some documented exceptions, most cancer cells do not possess or have defective primary cilium [105] (reviewed by Kiseleva et al. [106] and Eguether et al. [107]). This statement is further supported by multiple reports linking the hyperproliferative phenotype with the lack/reduction in ciliation [103,108] and supernumerary of centrioles [109]. While normal stem cells require cilia to maintain the quiescence state, many proliferative progenitors tend to lose cilia. Thus, it is critical to define the ciliation status of cancer stem cells, which supposedly originated from the normal stem cells.

Cancer stem cells (CSCs) or tumor-initiating cells (TICs) exhibit high levels of therapy resistance and the capability to repopulate the tumor after treatment [110,111,112]. However, a few mechanistic studies published in this field have shown that the effects of primary cilia on tumorigenesis and stemness are context-dependent.

Sonic hedgehog (Shh) signaling is cilia-dependent but not all cancers are Shh-dependent: One of the pioneering studies in this field came from Alvarrez-Buylla and colleagues on the origins of medulloblastoma [113]. Medulloblastoma is the most common brain cancer in children [114]. It is believed to arise from neural stem cell precursors [115] through aberrant activation of Shh signaling [115]. It was reported that ciliated precursor cells without Ptch1 were able to form medulloblastoma, while non-ciliated cells, even with constitutively active Smo protein, could not form tumors [113]. Hence, this study concludes that cilia are needed for tumorigenesis [116], suggesting that CSC or their progenitors might possess cilia. However, when the Hh pathway was dysregulated downstream of Smo/Ptch, cilia were exhibiting tumor-suppressive function [113]. The ciliated cells with mutant GliA could not form medulloblastoma because GliR proteins (also cilia activated) could balance the activity of the GliA. In this context, CSCs might be non-ciliated.

Further supporting this notion, Gate et al. identified a subset of medulloblastoma cells with stemness properties (CSC) that are characterized by high expression of Lewis X (CD15) protein. These cells were found more abundantly in recurrent medulloblastoma than in primary medulloblastoma. The recurrent cancers are treatment resistance and aggressiveness [117]. CD15+ medulloblastoma stem cells showed increased proliferation rates, expression of Hh response genes [118] and were not ciliated [117]. This data suggests that non-ciliated CSC might be more aggressive and resistant to treatment. Primary cilium was also tumor suppressive in granule cell progenitors-driven medulloblastoma due to localized ciliary Gpr161, a known inhibitor of Shh signaling [119]. In case of medullablastoma, presence or absence of primary cilia in precursor cells and how this affects the tumor formation depends on the step at which the Shh signaling cascade is dysregulated.

Similarly, rhabdomyosarcoma (RMS) and glioblastomas (GSCs) could be stratified as cilia/Hh-dependent and independent cases [25,120,121]. If RMS develops from undifferentiated myoblasts, which are ciliated, the cancer cells are Hh dependent, whereas if the development of RMS is from more differentiated progenitors, it is characterized by a lack of cilia, thus Hh independent. In general, the authors noted that ablation of primary cilium strongly suppresses Hh signaling while enhancing proliferation, while cilia rescue induces GSC differentiation and decreases proliferation [122,123]. In breast cancer, the decrease in ciliation in basal and luminal cells/progenitors has been well documented in patient biopsies [124] and mouse models [125,126]. Nevertheless, a few studies have reported the presence of rare ciliated Hh-dependent cells. These cells are cytokeratin (CK)5+, 6+, 15+ positive (basal progenitors) and might possess some stem cell features [127]. The Shh signaling is activated in CSCs of several malignancies [128] in the stromal compartment (reviewed here [129]), suggesting the paracrine regulation of Shh signaling in the mammary cancers. The presence of such cells (i.e., Shh activated) might explain an otherwise contradictory study on ciliated mammary stem cells (MaSCs) as a source of tumor-initiating cells [130].

Note that Shh-driven cancers might switch to other deciliation-dependent pathways during cancer progression, signifying limitations imposed by cilia. The examples include an inverse correlation between Ras/MAPK cascade activation and ciliation in basal cell carcinoma [131] and other K-Ras driven malignancies [132]. Authors suggest that primary cilium is an important lineage gatekeeper preventing Shh to Ras/MAPK switching. It is attractive to speculate that while the cilia-Hh axis is critical for normal stem cells, the Ras/MAPK might be attributed to deciliated CSCs.

Hypoxia—Driving Force of Stemness and Deciliation in Cancer Cells: The tumor microenvironment is critical in defining multiple aspects of cancer progression. In this regard, hypoxia is one of the key factors driving tumor dissemination, metastasis, epithelial-mesenchymal transition (EMT), and stemness. It was shown that hypoxic cancer cells and MSCs lose cilia in HIF1α, Wnt, TNFα, or NFκB-dependent manner [133,134]. von Hippel-Lindau disease tumor suppressor protein (VHL) targets HIF1α to proteosomal degradation in normal conditions. Note that hypoxia or mutation-driven inactivation of VHL stabilizes/activates AURKA [135,136], the key cilia disassembling factor. In summary, hypoxia might induce stemness and contribute to the deciliation of cancer cells through AURKA.

EMT is often observed in vitro in established cancer cells but rarely reported in clinical cancer biopsies. Moreover, EMT is not required for tumor initiation or growth but is primarily linked to dissemination. The induction of EMT in Biliary tree stem cells, the potential source of cholangiocarcinoma, leads to the opposite effect—loss of the cilium [137]; thus, the role of EMT in cilia biology might require further characterization.

Oncogenes and tumor suppressors are often altered during tumorigenesis via epigenetic changes. The Enhancer of Zest Homology 2 (EZH2) makes the methyltransferase subunit of the polycomb repressor complex 2 (PCR2) [138] and is considered to be an oncogene often activated in tumor cells. In melanoma, EZH2 suppresses the expression of ciliary biogenesis genes and drives the formation of metastatic melanoma with non-ciliated stem-like cells due to the increased activity of Wnt/β-catenin signaling [138]. The inactivation of EZH2 in glioma stem cells (GSCs) leads to the inability to generate neurospheres, which is indicative of the loss of stemness [139]. It can be speculated that the loss of stemness in GSCs is caused by increase in ciliation in these cells thorough increased expression of cilia biogenesis genes caused by inactivation of EZH2.

Intriguingly, the most prominent cancer stem cell marker, CD133/Prom1, was found to be a key regulator of ciliary dynamics and maintenance of the normal stem cell quiescence state [140]. The knockout of CD133 or overexpression of dominant-negative Prom1 mutant led to the loss of cilium [141]. Overexpression of CD133 in cancer stem cells may lead to sequestration of the components generally required for building primary cilium, thus inducing deciliation. In this context, the loss of stemness could be expected with the restoration of the cilia, but further studies in this field are needed to clarify the role of primary cilia in cancer stem cell biology and the effect of the tumor microenvironment.

## 12. Conclusions and Future Perspectives

The primary cilium is an essential organelle for stem cell development and differentiation. Several signaling pathways are interrupted if the formation of primary cilia is inhibited. The presence of functional primary cilium is essential during embryonic development, evident with the embryonic lethality of the whole body knock out of IFT88, KIF3A, and KIF3B in mouse models [36,37,38,39]. Furthermore, conditional depletion of primary cilia in adult stem cells results in aberrant signaling events [42,68,69,77,78,86,101]. Additionally, tissue-specific activation or inhibition of the Wnt/β-catenin pathway as a result of cilia depletion should also be noted. While cilium is a positive regulator of Wnt activation in osteal cells, it is the negative regulator of Wnt pathways in adipose, ESCs, and MEFs [68,69,71,72]. Similarly, the dual role of the primary cilium in signaling pathways can be seen in cancer stem cells [113]. Depletion or knockout of IFT88, KIF3A, OFD1 can be valuable tools to study the role of primary cilia in desired settings. It should be noted that, although lack or reduced quantities of these proteins will lead to the absence of primary cilia, the downstream impact of the elimination of primary cilia through the depletion of one or the other protein might be different. It is equally imperative to know the ramifications of stable and functional primary cilia as well as the precarious and malfunctioning one to characterize the essential role of this organelle in complex and dynamic systems like stem cells.

## 13. Clinical Relevance

- Understanding how the primary cilium is involved in stem cell biology is crucial to develop new ciliotherapies [25] to eliminate developmental ciliopathy diseases such as Meckel Syndrome.
- Targeting primary cilia and improving its stability/function in patients suffering from bone diseases might be a new approach to improve the patients’ quality of life.
- Deciphering the role of the primary cilium in cancer stem cell biology will likely improve our knowledge and diversify the cancer treatment options as the majority of the signaling pathways are dysregulated in cancers.

## 14. Outstanding Questions

- Does the knockout or depletion of essential ciliary genes such as IFT88, IFT20, KIF3A (“builders”) have the same impact on differentiation of the embryonic vs. adult stem cells or iPSCs? The answer to this question might allow for defining differences between signaling in dividing vs. quiescent stem cells.
- Since genes such as IFT88 and others have additional non-ciliary functions, the interpretation of KO results might be more complicated than just lack of cilia. Better tools need to be developed and expression controlled to decipher the cilium dependent vs. independent impact of the knockout of IFT and kinesin proteins.
- Would chemical inhibition of these proteins deliver the same results? The answer will allow us to discriminate between activity vs. protein-protein driven functions of the ciliary “builders” or “disassemblers”.
- Would the utilization of proteasome inhibitors in cilia deficient embryos recover the Notch (NICD) levels and HE to HPSC differentiation? The answer will be needed to understand the role of protein biosynthesis/degradation, including autophagy in ciliary homeostasis.
- Can we reprogram cancer stem cells via manipulation of primary cilia dynamics? The answer to this question will require substantial evidence to be gathered on the driving forces of deciliation in cancer, which includes but is not limited to centrosome defects/amplification, lack of cilia, ”builders”, or overexpression of cilia, “disassemblers”.

## Data Availability

Not applicable.
